# New Synthesis Route for Complex Borides; Rapid Synthesis of Thermoelectric Yttrium Aluminoboride via Liquid-Phase Assisted Reactive Spark Plasma Sintering

**DOI:** 10.1038/s41598-020-65818-z

**Published:** 2020-06-02

**Authors:** Hyoung-Won Son, David Berthebaud, Kunio Yubuta, Akira Yoshikawa, Toetsu Shishido, Keiko Suzuta, Takao Mori

**Affiliations:** 1 WPI International Center for Materials Nanoarchitechtonics (WPI-MANA), National Institute for Materials Science (NIMS), Namiki 1-1, Tsukuba 305-0044 Ibaraki, Japan; 2 Graduate School of Pure and Applied Sciences, University of Tsukuba, Tennoudai 1-1-1, Tsukuba, 305-8671 Ibaraki Japan; 3 CNRS-Saint Gobain-NIMS, UMI 3629, Laboratory for Innovative Key Materials and Structures (LINK), National Institute for Materials Science, Tsukuba, 305-0044 Ibaraki Japan; 4 Institute of Multidisciplinary Research for Advanced Materials, Tohoku University, 2-1-1, Katahira, Aoba-ku, Sendai, 980-8577 Miyagi Japan; 5 Nanotechnology Innovation Station, National Institute for Materials Science (NIMS), Namiki 1-1, Tsukuba 305-0044 Ibaraki, Japan

**Keywords:** Ceramics, Design, synthesis and processing, Thermoelectrics

## Abstract

Y_x_Al_y_B_14_ ceramics are of high interest as high temperature thermoelectric materials with excellent p, n control. In this study, direct synthesis of dense polycrystalline Y_x_Al_y_B_14_ (x ~0.64, 0.52 ≤ y ≤ 0.67) ceramics was successfully carried out by spark plasma sintering using commercially available precursors. YB_4_, AlB_2_ and B powders were reactively sintered with an additive AlF_3_ at 1773 K for 5–60 min in reduced Ar atmosphere. The sinterability was remarkably enhanced by liquid phase sintering comparing to conventional synthesis techniques. Phase composition analysis by X-ray diffraction showed that main peaks belong to Y_x_Al_y_B_14_ with the MgAlB_14_ structure type and no peaks of AlF_3_ were detected. The thermoelectric behavior was changed from p-type to n-type with increasing Al occupancy. Power factor and *ZT* values measured in this study were found to be in the same range as the best values previously reported. This original synthesis process is found to be less precursor-consuming as compared to previous synthesis processes, and strikingly, less time-consuming, as the synthesis time, is shortened from 8 h to 5 min for p-type and to 1 h for n-type. The total process time is shortened from ≥3 days to ~4–5 h. This discovery opens the door for more accessible synthesis of complex borides.

## Introduction

Thermoelectric materials utilize the solid state Seebeck effect and thereby can directly convert heat to electricity. This energy harvesting technique has some advantages such as reliability, simplicity, environmental-friendliness, and so on. Thermoelectric performance is determined by the dimensionless figure of merit, ${ZT}={S}^{2}\sigma T/\kappa$, where $S$ is Seebeck coefficient, $\sigma$ is electrical conductivity, $T$ is absolute temperature, and $\kappa$ is thermal conductivity. However, since $S$, $\sigma$ and $\kappa$ are basically in trade-off relationships, new approaches are required for breakthroughs^1–5^.

Recently, an important topic is to develop energy harvesting technologies to power IoT (Internet of Things) sensors and devices^6,7^. For such a goal, organic, organic-inorganic hybrid, and inorganic materials are being developed to use near room temperature as flexible or micro-sized thermoelectric power generation modules^8,9^.

On the other hand, at the other extreme of very high temperatures above 1000 K, there are various attractive high temperature thermoelectric applications. Namely, topping cycle for power plants, steelworks, industrial furnaces, etc^10–14^. In order to be used at such high temperatures, the materials need to possess robust high temperature stability. Suitable material groups for such applications are refractory ceramics like oxides^15–20^, nitrides^21,22^, and borides. For the borides, investigations have been particularly carried out on such boron icosahedral compounds such as boron carbide^23–27^, β-rhombohedral boron^28,29^, B_12_As_2_^30,31^, RB_44_Si_2_^32,33^, RB_66_^34,35^, MB_6_^36,37^, R-B-C (N) compounds^38–40^, and so on. One big issue for the borides is the difficulty of finding matching p-type and n-type compounds. The aforementioned compounds are predominantly p-type, with the exception of certain doped elements into beta-boron which have yielded n-type characteristic in a certain temperature range, and the R-B-C (N) compounds which are of interest as n-type counterparts to boron carbide^38–41^.

**Figure 1:**
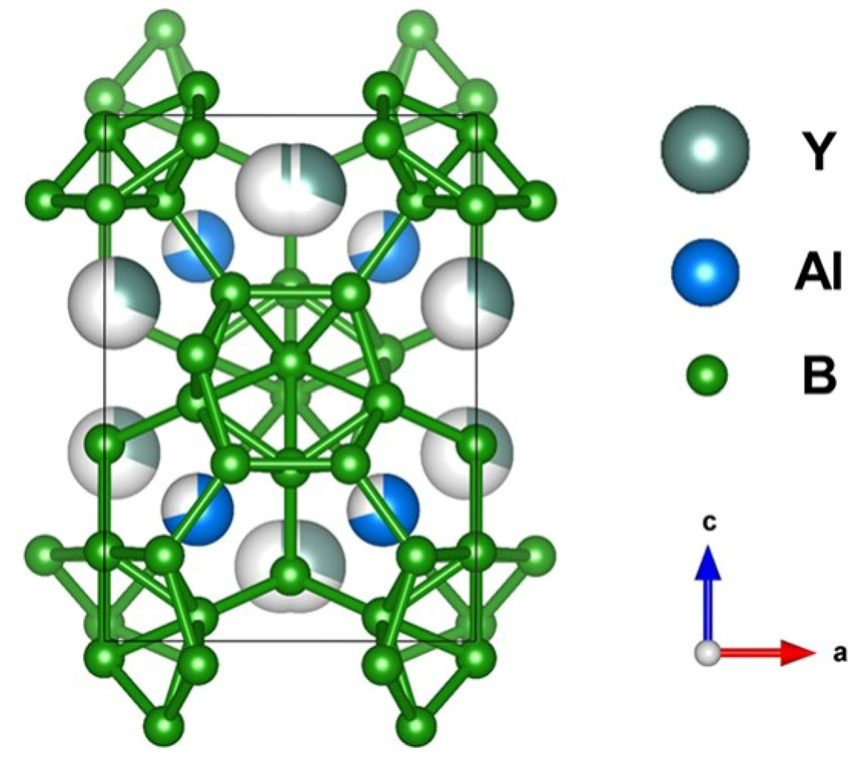
Depiction of crystal structure of Y_x_Al_y_B_14_, first reported by Korsukova *et al*.^42^, which is orthorhombic structure belonging to *Imma* space group. The boron atoms B1, B3, B4 and B5 form B_12_ icosahedra structures, which are interconnected via external B-B bonds or via B2 atom. Y and Al atoms partially occupy interstitial positions in the rigid three-dimensional B network.

Y_x_Al_y_B_14_with orthorhombic structure^42^, has been found to exhibit p- and n-type characteristics. Y and Al atoms partially occupy interstitial sites among B_12_ icosahedra clusters as shown Fig. 1. The thermoelectric behavior is changed from p-type to n-type by controlling Al occupancy. Y_x_Al_y_B_14_ also has high melting point and excellent hardness, and thus, it is expected to be a promising candidate for high temperature thermoelectric applications^41,43–46^.

Maruyama *et al*. and Sahara *et al*.^43–46^. have reported thermoelectric properties and microstructure of polycrystalline Y_x_Al_y_B_14_ ceramics with a various range of Al occupancy. Y_0.57_Al_y_B_14_ samples were synthesized with excess Al (y = 2.8–5.6) serving as flux, by sintering in induction furnaces for 8–12 h at 1400–1500 °C. To prepare dense bulk samples, the synthesized samples were pulverized, washed in NaOH solution, dried, and then sintered again by the spark plasma sintering (SPS) technique. SPS, which is one of the advanced sintering techniques, is effective for densification. In comparison with conventional sintering, SPS has some advantages such as rapid heating, shorter process time and densification at relatively lower temperatures^47–49^.

However, in spite of using SPS, relative densities of obtained n-type Y_x_Al_y_B_14_ (x = ~0.56; 0.57 ≤ y ≤ 0.63) samples exhibited quite low values (71.1–89.4%) due to the material’s poor sinterability. Furthermore, the previous synthesis method required a waste of resources by evaporation of large amounts of Al during the synthesis, and more importantly also requiring a complicated and time consuming total process^43–46^. This synthesis issue can be said to be a problem for many complex borides discovered with interesting electrical, magnetic, thermoelectric, and mechanical properties^50–54^. It is thus needed to develop a new, simple and reasonable synthesis process.

Recently, for synthesis and sintering of materials, which have poor sinterability, reactive SPS techniques and development of sintering additives have been studied. Son *et al*.^55^ reported about fabrication of translucent AlN by SPS with MgF_2_, which was used as a sintering additive. In general, AlN is also difficult to attain full densification due to its high covalent bonding. In that study, the liquid phase formed by MgF_2_ assisted sintering, and as a result, fully densified and translucent samples were obtained with short sintering time (20 min). Densification of complex borides which do not melt stably and have an upper limit on temperatures which can be used for sintering, are an even more difficult problem. For example, different sintering conditions^39,56^ and sintering aids were tried for the metal borocarbonitrides which are attractive thermoelectric materials, with some effective sintering aids found, but which were detrimental to the properties.

In this study, a new approach with reactive SPS is investigated to synthesize and prepare dense n-type Y_x_Al_y_B_14_ ceramics. As a new sintering additive, AlF_3_ is used. It is expected that AlF_3_ can enhance sinterability by forming a liquid phase during sintering due to its relatively low melting point.

## Results and Discussion

**Figure 2:**
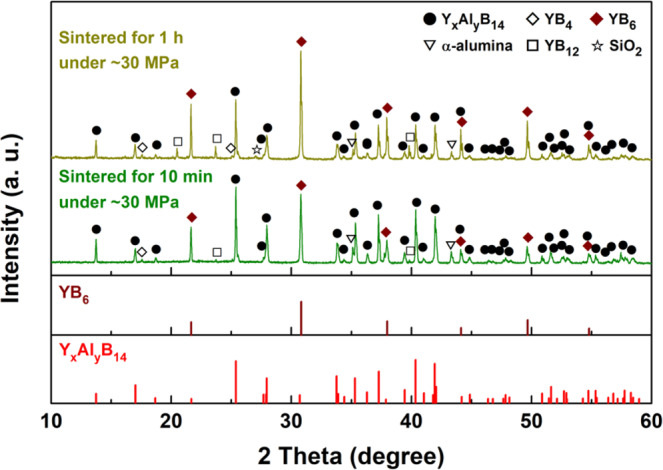
XRD patterns of YA0 composition (Y_0.62_Al_0.71_B_14_) samples prepared by reactive SPS without AlF_3_ addition. The samples were sintered at 1773 K under uniaxial pressure of ~30 MPa in a reduced Ar atmosphere for 1 h and 10 min, respectively. The standardized intensities for Y_x_Al_y_B_14_ and YB_6_ are shown with sample’s XRD patterns.

**Figure 3:**
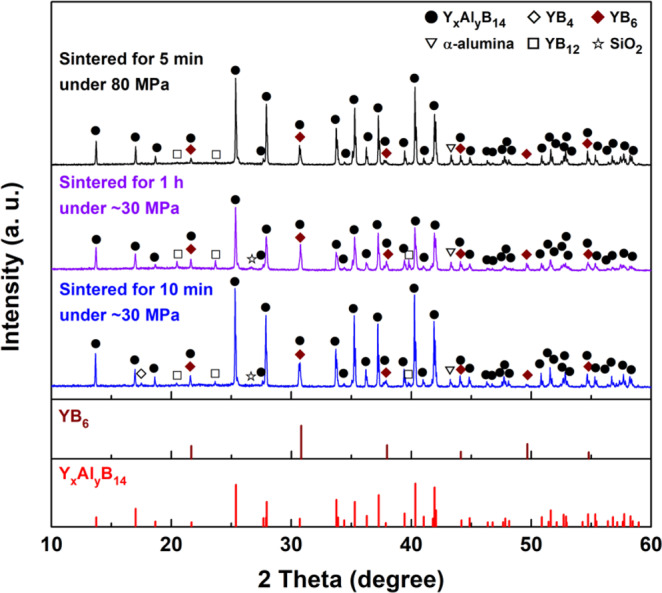
XRD patterns of YA0 composition (Y_0.62_Al_0.71_B_14_) samples prepared by reactive SPS with AlF_3_ addition. The samples were sintered at 1773 K in a reduced Ar atmosphere for 1 h and 10 min under the uniaxial pressure of ~30 MPa, and for 5 min under the uniaxial pressure of 80 MPa, respectively. The standardized intensities for Y_x_Al_y_B_14_ and YB_6_ are shown with sample’s XRD patterns.

Figures 2 and 3 show the XRD patterns measured on the surface of YA0 composition samples sintered with and without AlF_3_ at 1773 K, respectively. The XRD pattern of sample sintered for 10 min without AlF_3_ exhibits numerous peaks of secondary phases such as unreacted YB_4_, YB_6_, YB_12_, α-alumina, and so on, as shown in Fig. 2. The peaks of secondary phases are still detected on the YA0 composition sample which were even sintered for 1 h. It means that the reaction for synthesis of Y_x_Al_y_B_14_ is independent on sintering time in this case.

In contrast, the XRD patterns of the same composition samples sintered with AlF_3_ at the same sintering condition exhibit relatively much less peaks of secondary phases, and no peak of AlF_3_ was detected. It implies that AlF_3_ dramatically improved the reactivity among starting materials by liquid phase sintering, and then was completely removed without remaining by evaporation under the reduced Ar atmosphere. The relatively purest Y_x_Al_y_B_14_ was obtained for the sample sintered for 5 min under the uniaxial pressure of 80 MPa with AlF_3_ (Fig. 3). Although some peaks of α-alumina and YB_6_ phases are still detected as in the previous conventional synthesis studies^43,44^, polycrystalline Y_x_Al_y_B_14_ was successfully synthesized via reactive SPS with AlF_3_.

**Figure 4:**
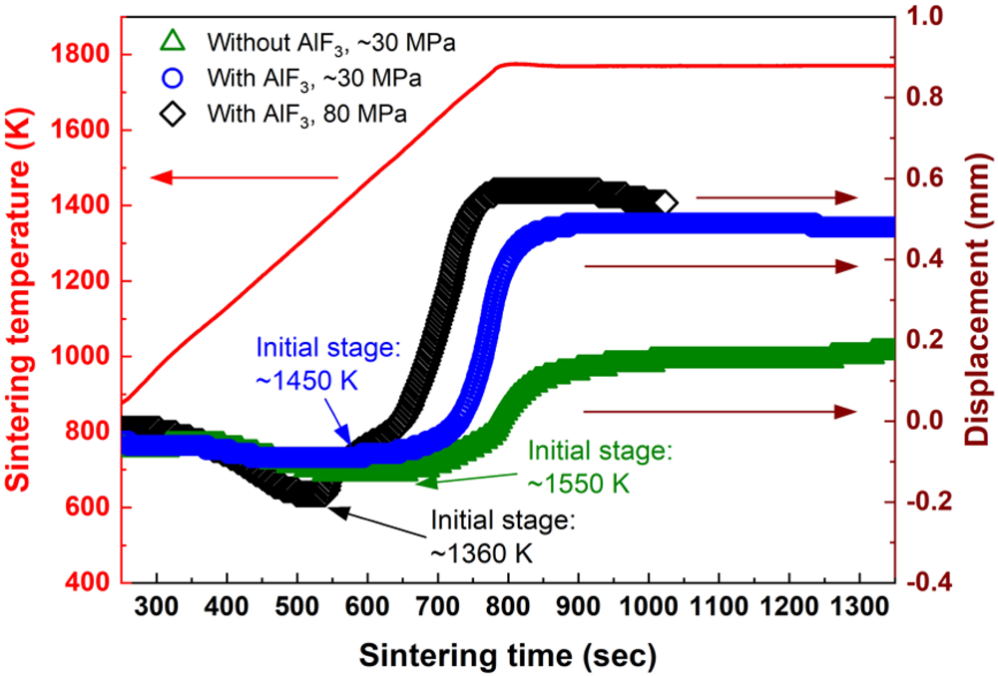
Densification behavior of Y_x_Al_y_B_14_ sintered without AlF_3_ under ~30 MPa, with AlF_3_ under ~30 MPa and with AlF_3_ under 80 MPa. To investigate the effect of AlF_3_ to densification, same amount of mixture and same graphite die and spacers were used.

The axial displacement changes of the YA0 composition samples during SPS are shown in Fig. 4. The temperature at the minimum displacement indicates the starting initial stage of sintering. In the case of the sample sintered without AlF_3_ under ~30 MPa which is the minimum pressure of SPS, the shrinkage was started at ~1550 K but densification was not finished even at 1773 K. On the other hand, in the case of the sample sintered with AlF_3_ under ~30 MPa, the shrinkage was started at ~1450 K and densified well during heating process. This result suggests that the sinterability and densification behavior were remarkably improved by AlF_3_. The melting point of AlF_3_ is ~1573 K at 1 atm. This temperature is higher than that for the starting shrinkage (~1450 K). It is therefore considered that the reduced-pressure Ar atmosphere lowered the melting point of AlF_3_, and the initial stage was shifted earlier, namely ~100 K lower by formation of liquid phase of AlF_3_.

The densification behavior of the YA0 composition sample sintered with AlF_3_ under uniaxial pressure of 80 MPa was further enhanced. The shrinkage of the pressure-assisted sample was started from ~1360 K, and the shrinkage rate was changed from ~1450 K. Since the temperature where shrinkage rate was changed is the same as the temperature of the starting initial stage of the sample sintered with AlF_3_ under ~30 MPa, the shrinkage observed from 1360 to 1450 K indicates the effect of the applied pressure. All samples exhibited changes of shrinkage rate during the intermediate stage. These phenomena can be mainly attributed to the different shrinkage rate between the main phase and the secondary phases.

**Figure 5:**
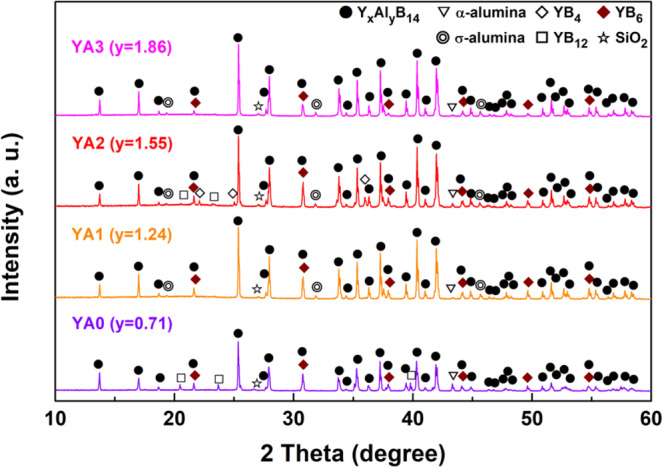
XRD patterns of YA0, YA1, YA2 and YA3 composition (Y_0.62_Al_y_B_14_; y = 0.71–1.86) samples prepared by reactive SPS with AlF_3_ addition. The samples were sintered at 1773 K under uniaxial pressure of ~30 MPa in a reduced Ar atmosphere for 1 h.

Figure 5 shows XRD patterns of Y_0.62_Al_y_B_14_ samples (YA0–3; y = 0.71–1.86) sintered at 1773 K with AlF_3_ for 1 h under ~30 MPa. Together with the prominent peaks of Y_x_Al_y_B_14_, weak peaks of several secondary phases such as α-alumina, σ-alumina, YB_4_, YB_6_, YB_12_ and SiO_2_ were detected. The Al-poor sample (YA0) exhibited peaks of α-alumina, YB_6_, YB_12_ and SiO_2_, whereas the Al-rich samples (YA1, YA2 and YA3) exhibited peaks of σ-alumina in addition. The structure of σ-alumina (Al_2.667_O_4_) is described as a defect cubic spinel in which Al cations randomly and partially occupy its octahedral and tetrahedral sites^57^. The result thus implies that addition of Al and reductive atmosphere affects formation of σ-alumina during sintering of Al-rich samples. For Al-rich samples, the intensity of the detected YB_6_ and YB_12_ peaks decreased with increasing Al content. It indicates that the excess Al compensated the Al loss caused by the formation of alumina phase during sintering. Relatively homogeneous yttrium aluminoborides were obtained for the YA3 composition. The XRD pattern for the YA2 sample shows the presence of YB_4_. It is attributed to inhomogeneous mixing of starting materials. To obtain Y_x_Al_y_B_14_ without secondary phases, such as SiO_2_, YB_4_, YB_6_ and YB_12_, optimization of the mixing process is required. We also found out that the SiO_2_ in the XRD patterns of the samples came from the agate mortar, which was used to pulverize each sample before powder XRD measurement. This was indicated because the intensity of the peak at 2*θ* = 26–27° increased with increasing pulverization time.

**Figure 6:**
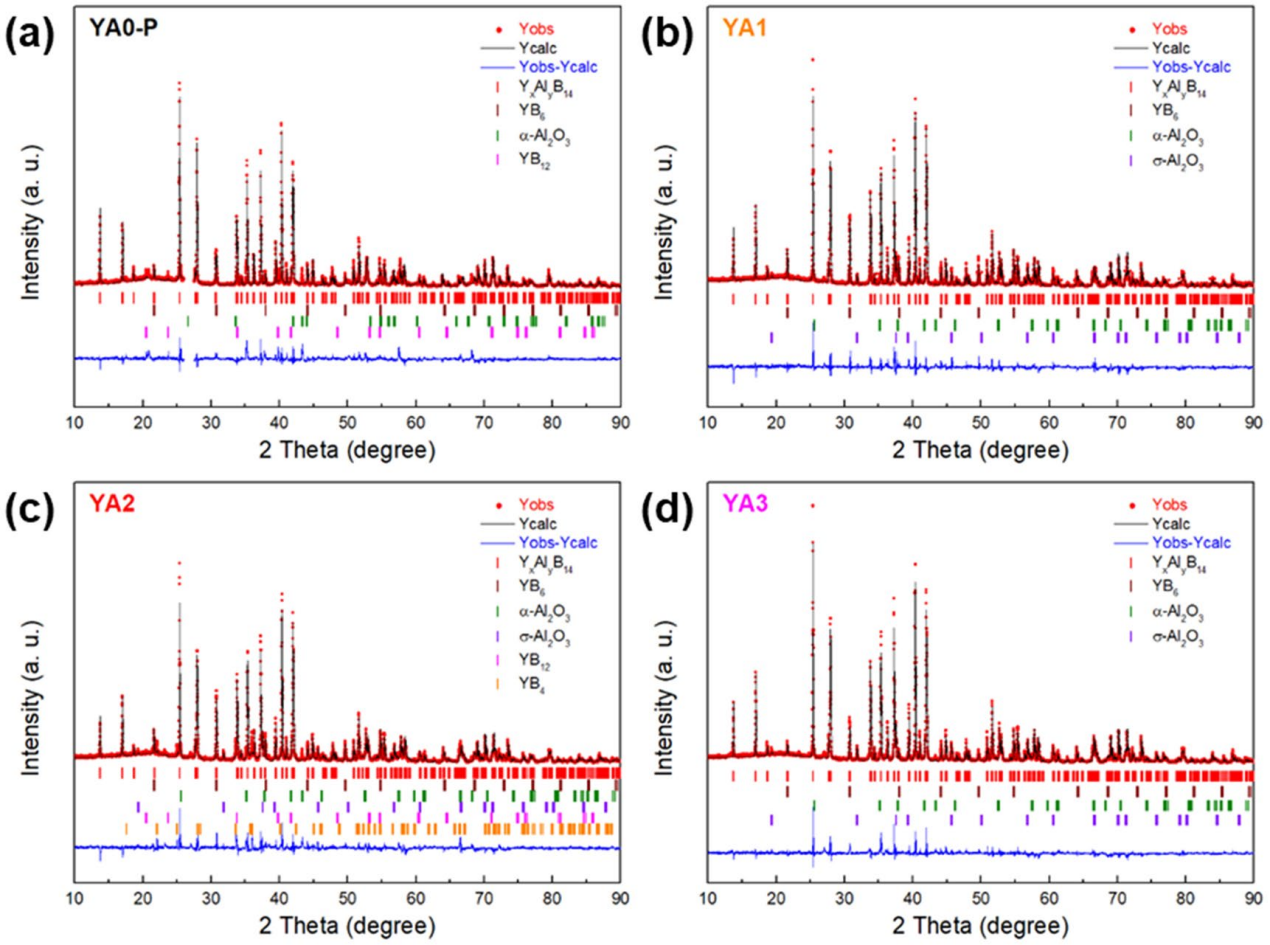
XRD pattern and the results of Rietveld refinement of YA0-P, YA1, YA2 and YA3 samples prepared by reactive SPS with AlF_3_ addition. The YA0-P sample was sintered at 1773 K for 5 min under 80 MPa, whereas the YA1, YA2 and YA3 samples were sintered at 1773 K for 60 min under ~30 MPa.

The fractional occupancies of x and y, of Y and Al, in the Al-poor (YA0-P) and -rich samples (YA1, YA2 and YA3) prepared by reactive SPS with AlF_3_ were estimated from Rietveld refinement within the technique limit (Fig. 6). At first starting atom position from Korsukova *et al*.^42^ model was used to perform refinements. Ultimately, atom positions were refined, together with partial occupancies for Y and Al positions. The synthesis conditions and the results of Rietveld refinement of each sample are summarized in Table 1 and Table 2, respectively. The refined Y contents (x in Y_x_Al_y_B_14_) of all samples are ~0.64, which is almost the same value as that of the starting materials. This result implies that no Y loss during reactive SPS occurred. The refined Al content (y in Y_x_Al_y_B_14_) of YA0-P is 0.52 though the starting content was 0.71. It suggests that Al was evaporated during sintering due to high sintering temperature and reduced Ar atmosphere. Meanwhile, the refined Al contents of YA1, YA2 and YA3 are ~0.67, which are much less than their initial Al contents (1.24, 1.55 and 1.86). Since these compositions are quite similar to those determined from single crystal x-ray diffractometry Y_0.62_Al_0.71_B_14_^42^, it is considered that the Y and Al atoms occupy their interstitial sites as maximumly as possible to satisfy the 4 electron deficient nature^53,54^, and thus excess Al could not occupy Al sites.

**Table 1:** The synthesis conditions of Y_x_Al_y_B_14_ samples.

Sample name	Starting composition	Sintering conditions (Temperature × time)	Pressure (MPa)	Atmosphere
YA0-P	Y_0.62_Al_0.71_B_14_	1773 K × 5 min	80	Ar
YA1	Y_0.62_Al_1.24_B_14_	1773 K × 60 min	~30	Ar
YA2	Y_0.62_Al_1.55_B_14_	1773 K × 60 min	~30	Ar
YA3	Y_0.62_Al_1.86_B_14_	1773 K × 60 min	~30	Ar

**Table 2:** Compositions and lattice parameters of sintered samples, which were estimated from Rietveld analysis.

Sample name	Starting composition	Refined composition	a (Å)	b (Å)	c (Å)	Volume of lattice (Å^3^)
YA0-P	Y_0.62_Al_0.71_B_14_	Y_0.66_Al_0.52_B_14_	5.825	10.401	8.205	497.107
YA1	Y_0.62_Al_1.24_B_14_	Y_0.64_Al_0.67_B_14_	5.812	10.416	8.190	495.805
YA2	Y_0.62_Al_1.55_B_14_	Y_0.62_Al_0.66_B_14_	5.811	10.414	8.188	495.503
YA3	Y_0.62_Al_1.86_B_14_	Y_0.65_Al_0.67_B_14_	5.811	10.413	8.189	495.516

The phase fractions and the theoretical and relative density of each sample are given in Table 3. At around 5%, the largest impurity phase is the alumina phase. We have tried to remove it but intrinsically find that it is difficult. As raw materials, amorphous boron is used for many sintering synthesis of complex borides which are difficult to synthesize by simple arc melting, for example^54^. There is some oxidation of the sensitive amorphous boron and in combination with aluminum for the present compound, alumina is formed. We tried to remove the oxygen by heating the amorphous boron and evaporation of BO, but found that handling in air, the amorphous boron is easily oxidized again. Therefore, in this case, the alumina impurity phase is intrinsically difficult to eliminate. However, together with metallic YB_6_, etc. which typically appear since mixing is generally not ideal, it is indicated that these secondary phases do not have a significant effect on the thermoelectric properties, as discussed below.

**Table 3:** Phase fraction of constituent phases and theoretical and relative density of each sample. The phase fraction of constituent phases and theoretical densities are estimated using Rietveld refinement.

Sample name	Y_x_Al_y_B_14_ (wt.%)	YB_6_ (wt.%)	α-Al_2_O_3_ (wt.%)	σ-Al_2_O_3_ (wt.%)	YB_12_ (wt.%)	YB_4_ (wt.%)	Theoretical density (g/cm^3^) (Relative density (%))
YA0-P	91.53	1.30	6.33	—	0.85	—	3.09 (96.1%)
YA1	92.65	2.52	0.16	4.67	—	—	3.08 (100%)
YA2	91.15	2.41	0.21	4.04	0.00	2.19	3.08 (98.7%)
YA3	93.07	0.89	0.15	5.89	—	—	3.08 (99.7%)

**Figure 7:**
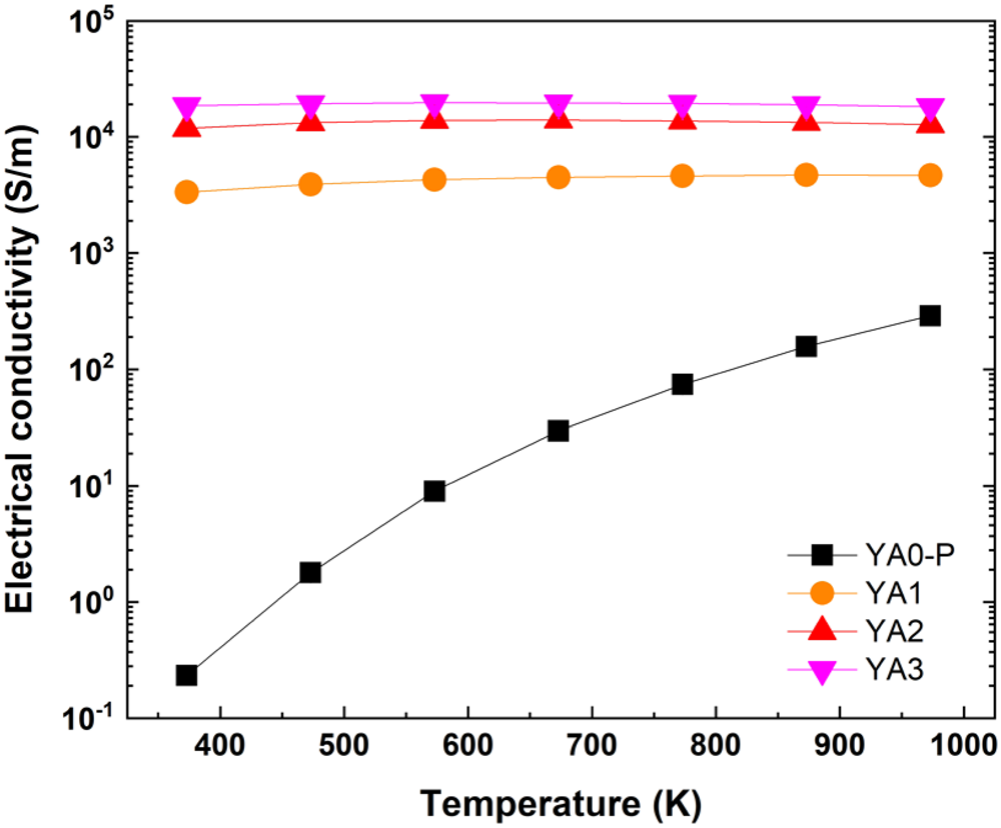
Temperature dependence of logarithmic electrical conductivities of Al-poor (YA0-P) and -rich samples (YA1, YA2 and YA3) prepared by reactive SPS. The YA0-P sample was sintered at 1773 K for 5 min under 80 MPa, whereas the YA1, YA2 and YA3 samples were sintered at 1773 K for 60 min under ~30 MPa. Error bars represent a measurement uncertainty range (±7%).

The temperature dependence of electrical conductivities for Al-poor (YA0-P) and -rich samples (YA1, YA2 and YA3) prepared by reactive SPS is shown in Fig. 7. At the low temperature region, the Al-poor sample exhibits extremely low electrical conductivity compared to Al-rich samples. However, a particularly large increase of electrical conductivity is observed with increasing temperature due to its conducting mechanism which will be described below. On the other hand, all the Al-rich samples exhibit several orders higher electrical conductivities. The highest electrical conductivity values are obtained for YA3 sample which is ~5 times higher than that of the YA1 sample. We consider the possibilities of the secondary phases having any effect on the thermoelectric properties. Amongst the thermoelectric properties, the electrical conductivity can be considered to have the potential to be most sensitive to small amounts of metallic phases or insulating phases, considering that these phases may have orders different resistivity than the main material. As an illustrative example we compare YA1 and YA3. From the Rietveld analysis, the composition (Table 2) and phase fraction (Table 3) of the main Y_x_Al_y_B_14_ phase is quite similar, as are the relative densities of the samples which are both close to 100% (Table 3). YA1 has a significantly larger amount of metallic YB_6_ impurities and also slightly less amount of insulating alumina impurities compared to YA3. However, as noted above, the conductivity of YA1 is actually around 5 times lower than YA3, indicating that the secondary phases in this case are not having a significant effect on the thermoelectric properties.

We consider the origin of the difference in properties. The large difference between YA1 and YA3 is only the starting composition (Table 2), where the amount of initial excess Al is around 1.5 times higher for YA3. Since Al itself cannot be seen in the XRD patterns, we speculate that Al atoms may be seeded in the grain boundaries and result in an enhancement of the electrical conductivity. Such an effect has been proposed before^58^ and observed in Ba seeding of Ca_3_Co_4_O_9_ for example^59^.

**Figure 8:**
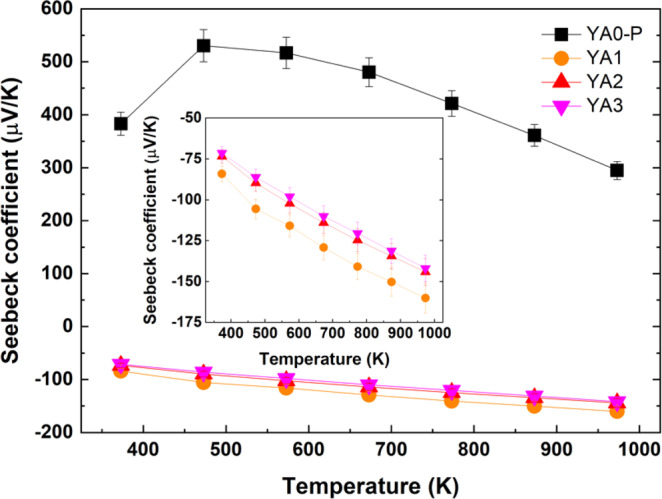
Temperature dependence of Seebeck coefficients of Al-poor (YA0-P) and -rich samples (YA1, YA2 and YA3) prepared by reactive SPS. The YA0-P sample was sintered at 1773 K for 5 min under 80 MPa, whereas the YA1, YA2 and YA3 samples were sintered at 1773 K for 60 min under ~30 MPa. The Seebeck coefficients of Al-rich samples are shown in the inset. Error bars represent a measurement uncertainty range (±7%).

Figure 8 shows the temperature dependence of Seebeck coefficients for each sintered sample. The Seebeck coefficient of Al-poor sample exhibits positive values, whereas those of Al-rich samples exhibit negative values. This indicates that the thermoelectric behavior of Y_x_Al_y_B_14_ samples are changed from p-type to n-type with increasing Al occupancy. The Seebeck coefficient values for Al-poor sample increases up to 473 K, and then decreases with increasing temperature. In contrast, the absolute values of negative Seebeck coefficients for Al-rich samples increase with increasing temperature. The maximum positive and negative values, 530 μV/K and −160 μV/K, are obtained for YA0-P at 473 K and for YA1 at 973 K, respectively.

**Figure 9:**
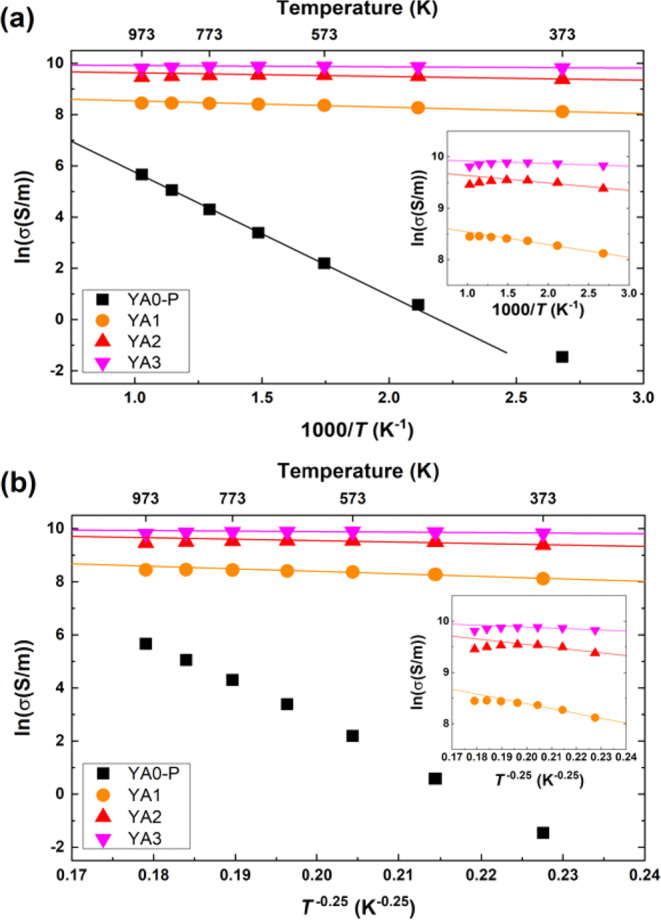
Temperature dependence of electrical conductivity of each sintered sample. The ln*σ* is plotted versus (**a**) *T*^-1^ for TAC mode and (**b**) *T*^-0.25^ for VRH mode, respectively. The straight line for Al-poor sample in TAC mode expresses the fit to Eq. 1 for the temperature range from 573 K to 973 K. Also, the straight lines for Al-rich samples in TAC mode and VRH mode express the fit to Eq. 1 and to Eq. 2 for the temperature range from 373 K to 673 K, respectively.

Figure 9(a,b) show the temperature dependence of electrical conductivity of each sintered sample in accordance with thermally activated conduction (TAC) mode and variable range hopping (VRH) mode, respectively. According to the TAC model, the conductivity is expressed as the following;

1 $${{\rm{\sigma }}}_{{\rm{TAC}}}={\sigma }_{0,{\rm{TAC}}}\exp (\,-\frac{{E}_{A}}{kT})$$

Meanwhile, in disordered systems, according to the VRH model, the conductivity for three dimensional systems is given by

2 $${{\rm{\sigma }}}_{{\rm{VRH}}}={\sigma }_{0,{\rm{VRH}}}\exp {\left(-\frac{{T}_{0}}{T}\right)}^{\frac{1}{4}}$$

where ${\sigma }_{0,{\rm{TAC}}}$ and ${\sigma }_{0,{\rm{VRH}}}$ are constants which do not depend on temperature, ${E}_{A}$ is the thermal activation energy, ${T}_{0}$ is the characteristic temperature determined by the density of localized states near the Fermi level, $k$ is the Boltzmann’s constant and $T$ is the absolute temperature^60^.

We consider the validity of the two different conduction models for our samples. In Fig. 9(a), the temperature dependence of the electrical conductivity of the Al-poor sample appears to generally follow the TAC dependence, except it appears to deviate from a straight line below 573 K. In contrast, in Fig. 9(b), the temperature dependence of the electrical conductivity appears to not be as good a fit to the VRH dependence as it shows a curvature over a wider temperature range. This implies that TAC is the dominant conduction mechanism for Al-poor Y_x_Al_y_B_14_ above 573 K. In this case, the calculated ${E}_{A}$ and ${\sigma }_{0,{\rm{TAC}}}$ from the fitted line are 0.4 eV and 3.95 × 10^4^ S/m, respectively. This activation energy value is similar to the band gap energy in the result of band structure calculation for Y_0.50_Al_0.75_B_14_^45^.

We will consider the mechanism for the low temperature deviation below together with the discussion on the Seebeck coefficient. For Al-rich samples, neither the TAC (Fig. 9(a)) or VRH (Fig. 9(b)) is a good fit, with the rate of increase of the electrical conductivity apparently suppressed at higher temperatures. We speculate that the reason of this suppression may be due to the presence of the Al segregation. This effect only appears to be noticeable for the highest conductive samples (i.e. Al-rich samples at high temperatures) and does not appear to be pronounced for the Al-poor sample where the level of electrical conductivity is much lower to begin with. For the Al-rich samples we attempt to obtain insight on the conduction mechanism through the temperature dependence of the Seebeck coefficient discussed below.

**Figure 10:**
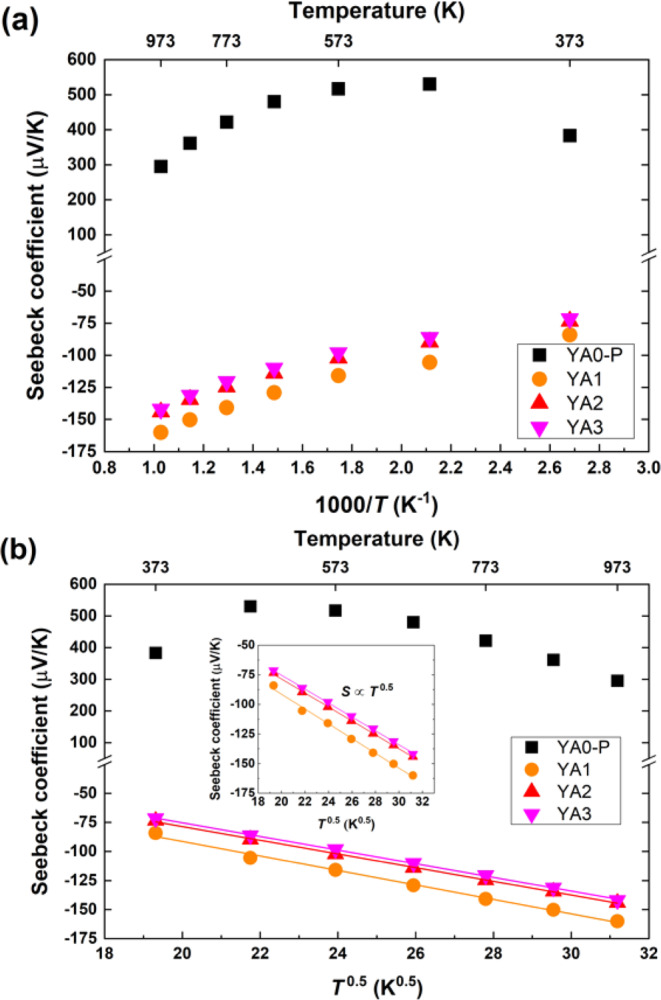
Temperature dependence of the Seebeck coefficients of all samples in accordance with (**a**) TAC mode and (**b**) VRH mode. The inset shows the Seebeck coefficients of Al-rich samples in accordance with VRH mode. The straight lines for Al-rich samples in VRH mode express the fit to Eq. 5 for the temperature range from 373 K to 973 K.

The temperature dependences of Seebeck coefficients for TAC and VRH are shown in Fig. 10. When the carrier concentration is increased by thermal activation, the Seebeck coefficient is generally expressed as the following^61,62^;

3 $${\rm{S}}=-\left(\frac{k}{e}\right)\left(\frac{{E}_{s}}{kT}+A\right)$$

where *e* is carrier charge, *k* is Boltzmann constant, *E*_*s*_ is the characteristic carrier-generation energy, $T$ is the absolute temperature, and $A$ is the heat-of-transport constant. However, the plots of Seebeck coefficients of all samples do not appear to have a clear *T*^-1^ component, not only for n-type but also for p-type Y_x_Al_y_B_14_ (Fig. 10(a)) which generally follows TAC for the electrical conductivity as discussed above.

The reason can be elucidated by considering the mixed conduction case in semiconductors. In that case, the total Seebeck coefficient is given by^61^

4 $$S=\frac{({S}_{n}{\sigma }_{n}+{S}_{p}{\sigma }_{p})}{({\sigma }_{n}+{\sigma }_{p})}$$

where ${\sigma }_{n}$ and ${\sigma }_{p}$ are the electrical conductivity due to electrons and holes, and ${S}_{n}$ and ${S}_{p}$ are the Seebeck coefficient for n-type and p-type conduction, respectively. Considering the temperature dependence of electrical conductivity discussed above, namely, the deviation to higher electrical conductivity at higher temperatures, the decrease of Seebeck coefficient of the Al-poor YA0-P above 573 K can therefore be attributed to the increase of thermally excited electrons.

In VRH systems, the temperature dependence of the Seebeck coefficient can be approximated by the following^62^;

5 $${\rm{S}}\propto {({T}_{0}T)}^{\frac{1}{2}}\left(\frac{{\rm{dln}}N(E)}{{\rm{d}}E}\right){|}_{E={E}_{F}}$$

where N(E) is the density of states at the Fermi level. For Al-rich samples, the temperature dependence of the Seebeck coefficients is almost proportional to *T*^0.5^, as shown in Fig. 10(b). From this temperature dependence of the Seebeck coefficient it is indicated that the Al-rich samples are VRH systems, since insulating or metallic impurities have less effect on the Seebeck coefficient.

To summarize what we have concluded from the data, the conduction mechanism of the Al-poor sample appears to follow TAC dependence, with deviations in the temperature dependences above 573 K indicated from thermally excited electrons. With the large increase of Al inserted atoms, and thereby electrons, for the Al-rich samples, not to mention a speculated effect of excess segregated Al atoms for YA3 in particular, the magnitude of electrical conductivity increases greatly compared to the Al-poor sample. However, despite this, it is indicated that the conduction mechanism follows the VRH mechanism, indicating that the electrical carriers have some localization due to disorder.

**Figure 11:**
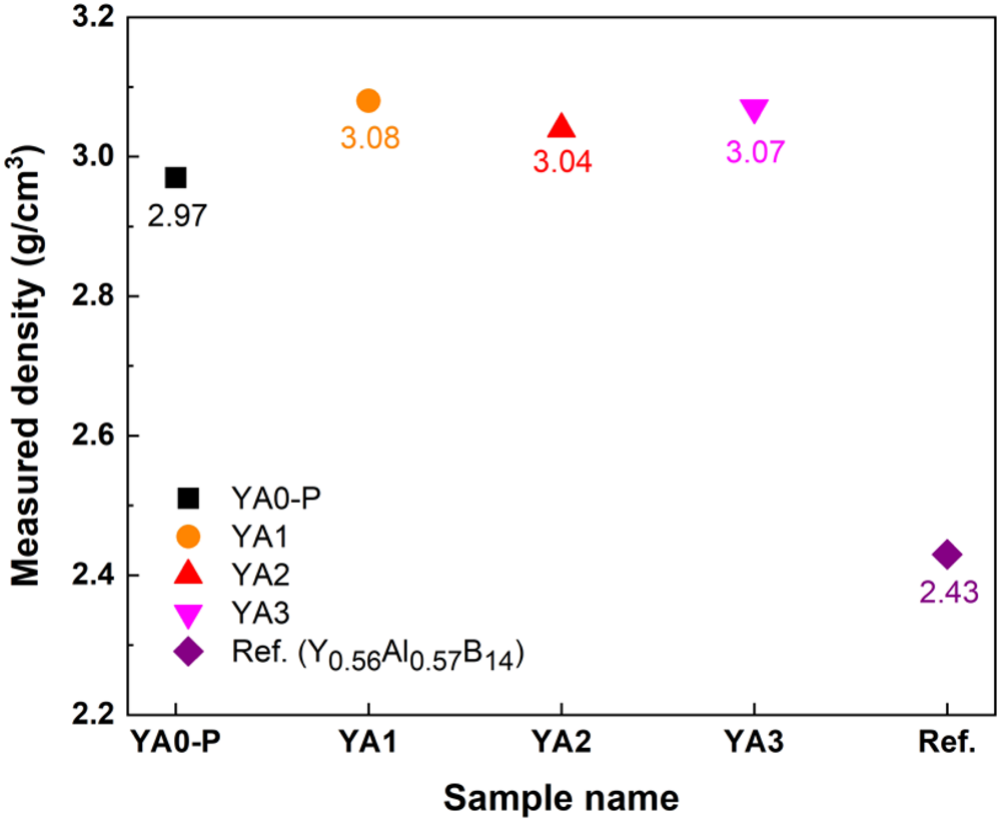
Measured densities of as-sintered Y_x_Al_y_B_14_ (x~0.64) samples, which are compared with the reported value of Y_0.56_Al_0.57_B_14_^44^. The theoretical and relative densities of the samples prepared by reactive SPS in the present study are given in Table 3.

Figure 11 shows the densities of the sintered bodies measured by Archimedes’ method. All samples prepared by reactive SPS exhibit rather high densities, 2.97–3.07 g/cm^3^, compared to that of the reference sample (2.43 g/cm^3^)^44^, and are close to the theoretical density of the single crystal Y_0.62_Al_0.71_B_14_, 3.02 g/cm^3^. The measured densities for Al-rich samples are a little bit higher than the reported theoretical density of single crystal due to the presence of secondary phases. However, even taking into consideration the presence of a relatively small amount of secondary phases, highly dense samples were obtained (Table 3). This result suggests that the new synthesis process via reactive SPS with AlF_3_ is also much more effective for densification of yttrium aluminoboride than the conventional process.

**Figure 12:**
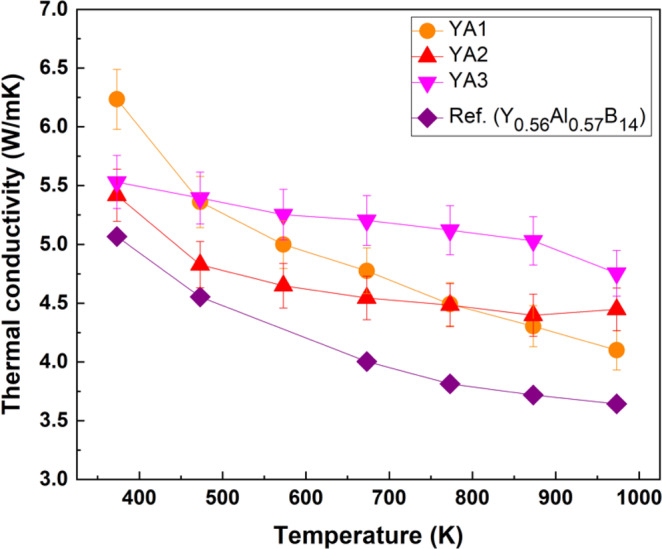
Thermal conductivities of n-type Y_x_Al_y_B_14_ (x ~0.64) samples prepared by reactive SPS, which are compared with the reported value of Y_0.56_Al_0.57_B_14_^44^. Error bars represent a measurement uncertainty range (±5%).

The thermal conductivities of n-type Y_x_Al_y_B_14_ samples are shown in Fig. 12. Thermal conductivities generally decrease with increasing temperature indicating Umklapp scattering. YA2 and YA3 show some difference in the temperature dependence, with relatively less temperature dependence compared to YA1 and YA0-P. This trend is also reflected to a lesser extent in the intrinsic thermal diffusivities (Fig. S1). The behavior may be due to the Al segregation speculated before, since both YA2 and YA3 have higher Al starting compositions, but it is not clear at present. In comparison to reported samples, all samples exhibit slightly higher thermal conductivities due to their relatively higher densities than what has previously been obtained.

**Figure 13:**
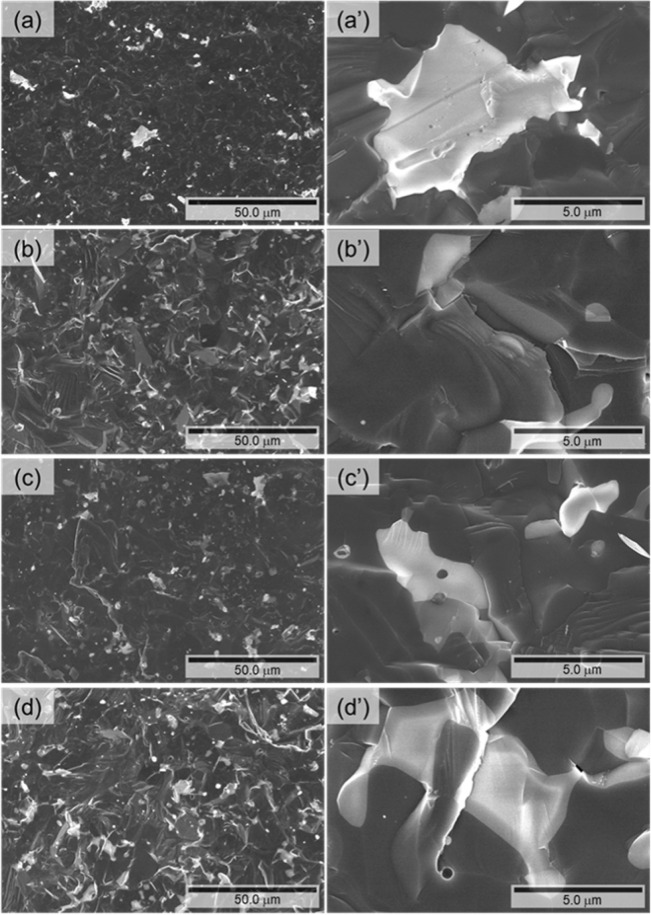
SEM images of fracture surface for Y_x_Al_y_B_14_ samples prepared by reactive SPS, (**a,a’**) YA0-P, (**b,b’**) YA1, (**c,c’**) YA2 and (**d,d’**) YA3.

**Figure 14:**
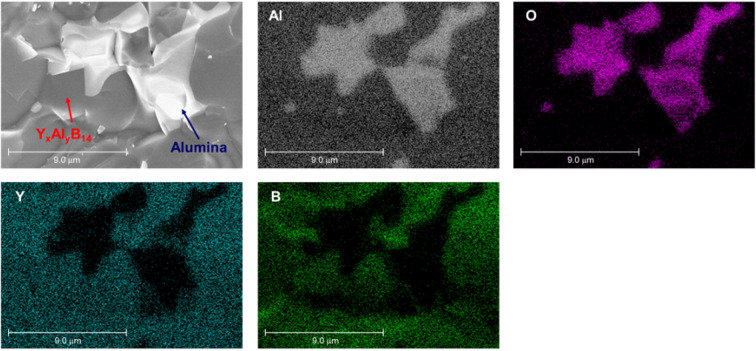
The result of EDX analysis on the microstructure in fracture surface of YA1. The distribution of Y, Al, B and O atoms were investigated by the mapping technique.

**Figure 15:**
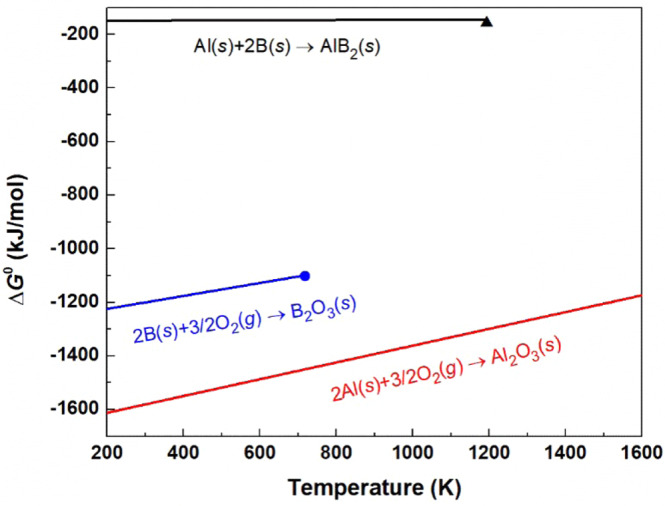
Gibbs free energy of formation of AlB_2_, B_2_O_3_ and Al_2_O_3_ with temperature range. The point positioned at the end of B_2_O_3_ line indicates melting temperature of B_2_O_3_ (723 K), and the triangle positioned at the end of AlB_2_ line indicates decomposition temperature (~1193 K)^64^.

To investigate the microstructure, SEM observation for fracture surface of the samples was carried out (Fig. 13). The samples synthesized by reactive SPS exhibit highly dense microstructure compared to that of previous samples^46^ though a minor amount of small pores are observed. All samples show coarse microstructure due to the high sintering temperature and liquid phase introduced during sintering. The microstructures generally consist of two phases, which are observed as a dark region and a bright region. The components in both regions are revealed by EDX analysis, as shown in Fig. 14. The result shows the presence of main elements, Y, Al, B and O. The dark region contains Y, Al and B but not O, whereas the bright region contains Al and O but not Y and B. It thus can be considered that the matrix and the secondary phase are Y_x_Al_y_B_14_ and alumina, respectively. In the matrix phase, some differences in the contrast are observed. It implies an inhomogeneity of the metal atoms which occupy the interstitial sites in Y_x_Al_y_B_14_. Standard molar enthalpy of formation ($\triangle {H}_{f}^{0}$) and standard molar entropy (*S*^0^) of each material are listed in Table 4 ^63^. The calculated free energies of formations (∆*G*^0^) for AlB_2_, Al_2_O_3_ and B_2_O_3_ were listed and plotted, as shown in Table 5 and Fig. 15^64^. The results of thermodynamic calculations show that ∆*G*^0^ of α-alumina is much lower than AlB_2_ and B_2_O_3_. As discussed previously, it is considered that the presence of boron oxide layer coated on the surface of particles of the starting material of amorphous boron reacted with Al during sintering, and thus the alumina phase was formed. We have found that even after removing the boron oxide by the pre-treatment of heating, the amorphous boron powder can have oxidation in air again. To suppress the formation of alumina phase, using a closed system to enable heating of the raw material amorphous boron and then continuously, the mixing process and SPS sintering is desirable.

**Figure 16:**
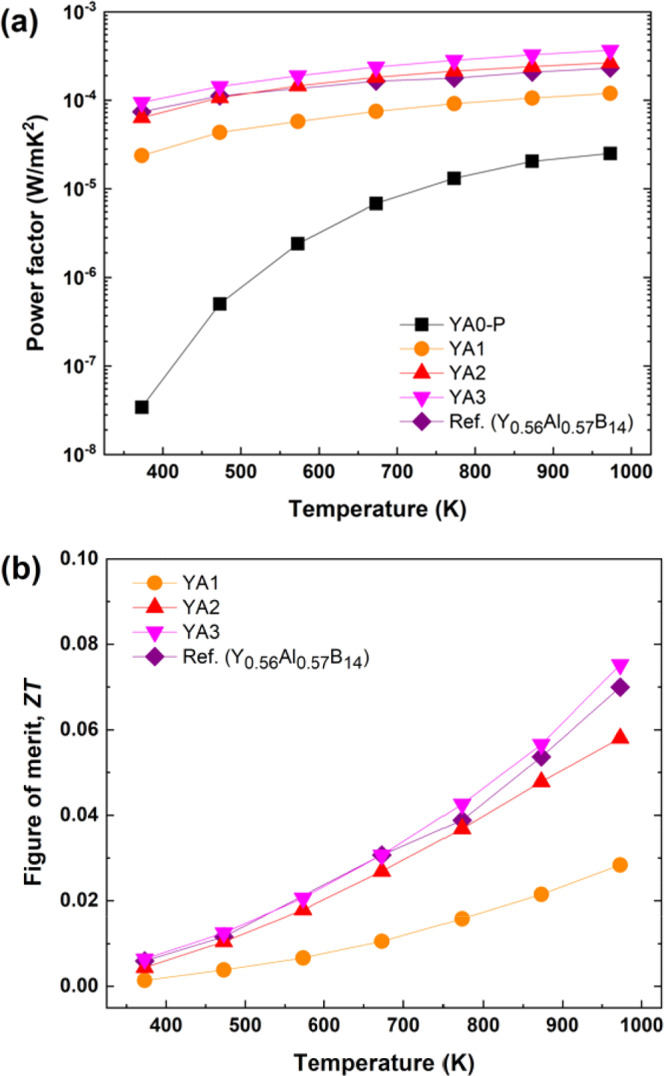
(**a**) Power factor and (**b**) figure of merits for Y_x_Al_y_B_14_ (x ~0.64) samples prepared by reactive SPS, which are compared with the reported value of Y_0.56_Al_0.57_B_14_^44^.

Figure 16(a,b) show the power factors and *ZT* values of Y_x_Al_y_B_14_ samples with the reference sample^44^, respectively. The power factor is dramatically improved by increasing Al occupancy. The maximum power factor, 3.7 × 10^−4^ W/mK^2^, was obtained for YA3 at 973 K, which is significantly higher than that of the reference Y_0.56_Al_0.57_B_14_ sample(Fig. 16(a)). The large improvement in power factor can be attributed to the high density achieved by the new synthesis process. The maximum *ZT* value, 0.08 was also obtained for YA3, as shown in Fig. 16(b). This value is comparable with those reported by previous study, because of the higher thermal conductivity. However, looking forward, it can be said that in general it is more straightforward to find ways to selectively lower the thermal conductivity^65,66^, rather than enhance the power factor^67,68^, Although the *ZT* is not high, there are very few materials which potentially can be used for very high temperature thermoelectric applications^10^, and we succeeded to prepare Y_x_Al_y_B_14_ by developing a new sintering process, which is much shorter, simpler and more cost-effective.

**Table 4:** Standard molar enthalpies of formation and standard molar entropies at 298 K^63^.

Material	$\Delta {H}_{f}^{0}$ (kJ/mol)	S^0^(J/molK)
O_2_ (*g*)	0	205.15
B (*s*)	0	5.9
Al (*s*)	0	28.3
Al_2_O_3_ (*s*)	−1675.7	50.92
AlB_2_ (*s*)	−151	34.7
B_2_O_3_ (*s*)	1273.5	53.97

**Table 5:** Gibbs free energy of formation changes accompanied by each reactions.

Reaction	Gibbs free energy of formation (J/mol)
Al(*s*) + 2B(*s*) → AlB_2_(*s*)	∆*G*^0^ = −151 + 0.0054 *T*
2Al(*s*) + 3/2O_2_(*g*) → Al_2_O_3_(*s*)	∆*G*^0^ = −1675.7 + 0.3134 *T*
2B(*s*) + 3/2O_2_(*g*) → B_2_O_3_(*s*)	∆*G*^0^ = −1273.5 + 0.2420 *T*

## Conclusions

In this study, a new synthesis process with SPS and sintering additive for synthesis of dense yttrium aluminoboride was developed. The addition of 1 wt.% AlF_3_ effectively and dramatically enhanced the sinterability and densification behavior of yttrium aluminoboride by the formation of liquid phase during SPS. Consequently, the process was remarkably simplified and total process time was shortened from ≥3 days to only ~4–5 h.

For p-type Y_x_Al_y_B_14_, the thermally activated conduction mechanism was proposed rather than the usually assumed variable range hopping (VRH) mechanism, to explain the experimental results of the temperature dependence of electrical conductivity and Seebeck coefficient. Conversely, the VRH mechanism appears to be a good match for the observed properties of the n-type Y_x_Al_y_B_14_. Because of the increasing Al occupancy and thereby electron carrier content, the n-type samples have much higher conductivity, however, despite this it is indicated that the electrical carriers have some localization due to disorder. *ZT* values of yttrium aluminoboride prepared by reactive SPS exhibits overall similar thermoelectric properties as the previously reported best samples prepared by conventional processes. The power factor is significantly enhanced because of the high density achieved by the present discovered method. The higher thermal conductivity balances out the *ZT*, however, in thermoelectric research there are many methods to selectively lower the thermal conductivity. In any case, the present synthesis method that we discovered radically shortens the time necessary for synthesis, e.g. from 8 h to 5 min for the p-type sample. This breakthrough technique is expected to be able to be applied to synthesize other borides also.

## Methods

Commercially available YB_4_ (99.9%, Japan New Metals Co., Ltd.), AlB_2_ (Sigma-Aldrich Co., Ltd.), amorphous B (99%, New Metals and Chemicals Co., Ltd.) and AlF_3_ (Wako Co., Ltd.) powders were used as the starting materials. YB_4_ and AlB_2_ were selected as the precursor for reactive sintering, and AlF_3_ was selected as the sintering additive. The powders of YB_4_, AlB_2_, B and 0–1 wt.% AlF_3_ were simply mixed under ethanol for 30 min using SiO_2_ mortar with nominal compositions of Y_0.62_Al_y_B_14_, where y = 0.71, 1.24, 1.55 and 1.86. For y = 0.71 (YA0 samples), five different sintering conditions were tested;10 min and 1 h under the uniaxial pressure of ~30 MPa, each with and without AlF_3_, and 5 min under the uniaxial pressure of 80 MPa with AlF_3_. Subsequent samples YA1, YA2 and YA3 were prepared with AlF_3_ additive and 1 h sintering time. The excess amount of Al (y = 1.24–1.86) was used to prepare Al-rich n-type yittrium aluminoborides.

The powder mixtures were dried at 373 K for 3 h in an oven, and then poured into a 10 mm diameter graphite die. Here, graphite paper was used as a release agent. The mixtures were heated up to 1773 K in a SPS apparatus (Dr. Sinter, Fuji Denpa Koki Co., Ltd.) with heating rate of 100 K/min, and then reactively sintered at 1773 K for 5–60 min under uniaxial pressure of 28–80 MPa in a reduced-pressure Ar atmosphere (−0.03 MPa). After sintering, the samples were cooled down with cooling rate of 200 K/min, and finally, the sintered discs, 10 mm in diameter and ~2 mm in thickness, were obtained.

After sintering, as-sintered samples were slightly grinded by a diamond plate (#400) to remove the graphite paper on their surface. The densities of grinded samples were measured using Archimedes’ method. The constituent phases of sintered samples were analyzed by X-ray diffraction (XRD, Smart lab 3, Rigaku) with Cu Kα radiation (λ=1.5418 Å). The XRD patterns were collected at 2*θ* = 10–90° with in a step of 0.01°. The compositions of the prepared Y_x_Al_y_B_14_ samples by reactive SPS were estimated from Rietveld refinement using FullProf software. The thermal diffusivity of each sample was measured from 373 to 973 K by using a laser flash analysis apparatus (LFA, TC-7000, ADVANCE RIKO, Inc.). Prior to the LFA, the samples were coated with carbon. After LFA, samples were cleaned to remove the carbon on their surfaces, and then machined into bar-shaped specimens by cutting machine. The electrical resistivity and Seebeck coefficient of the specimens were evaluated from 373 to 973 K using a thermoelectric tester (ZEM-2, ADVANCE RIKO, Inc.). The distance between probes used for the measurements was 5.6 mm. The specific heat capacities of the samples were measured from 313–1073 K using a high temperature differential scanning calorimetry (STA449F3-NT24, NETZSCH Japan Co., Ltd.). As crucibles, Pt pans, Pt lids and Alumina liner were used. Microstructure of fracture surfaces of each sample was observed by scanning electron microscopy (SEM, S-4800, Hitachi High-Technologies Co.) at an acceleration voltage of 10.0 kV. To investigate the components of matrix and secondary phases on the fracture surface, energy-dispersive X-ray spectroscopy (EDX, Hitachi High-Technologies Co.) was used.

## Supplementary information

Supplementary information.
