# Supplementary material for: New Synthesis Route for Complex Borides; Rapid Synthesis of Thermoelectric Yttrium Aluminoboride via Liquid-Phase Assisted Reactive Spark Plasma Sintering
Source: Sci Rep. 2020 Jun 2;10:8914. doi: 10.1038/s41598-020-65818-z (PMC7265475; doi:10.1038/s41598-020-65818-z)
Supplement: Supplementary file 1 — Supplementary information. [file 41598_2020_65818_MOESM1_ESM.docx]

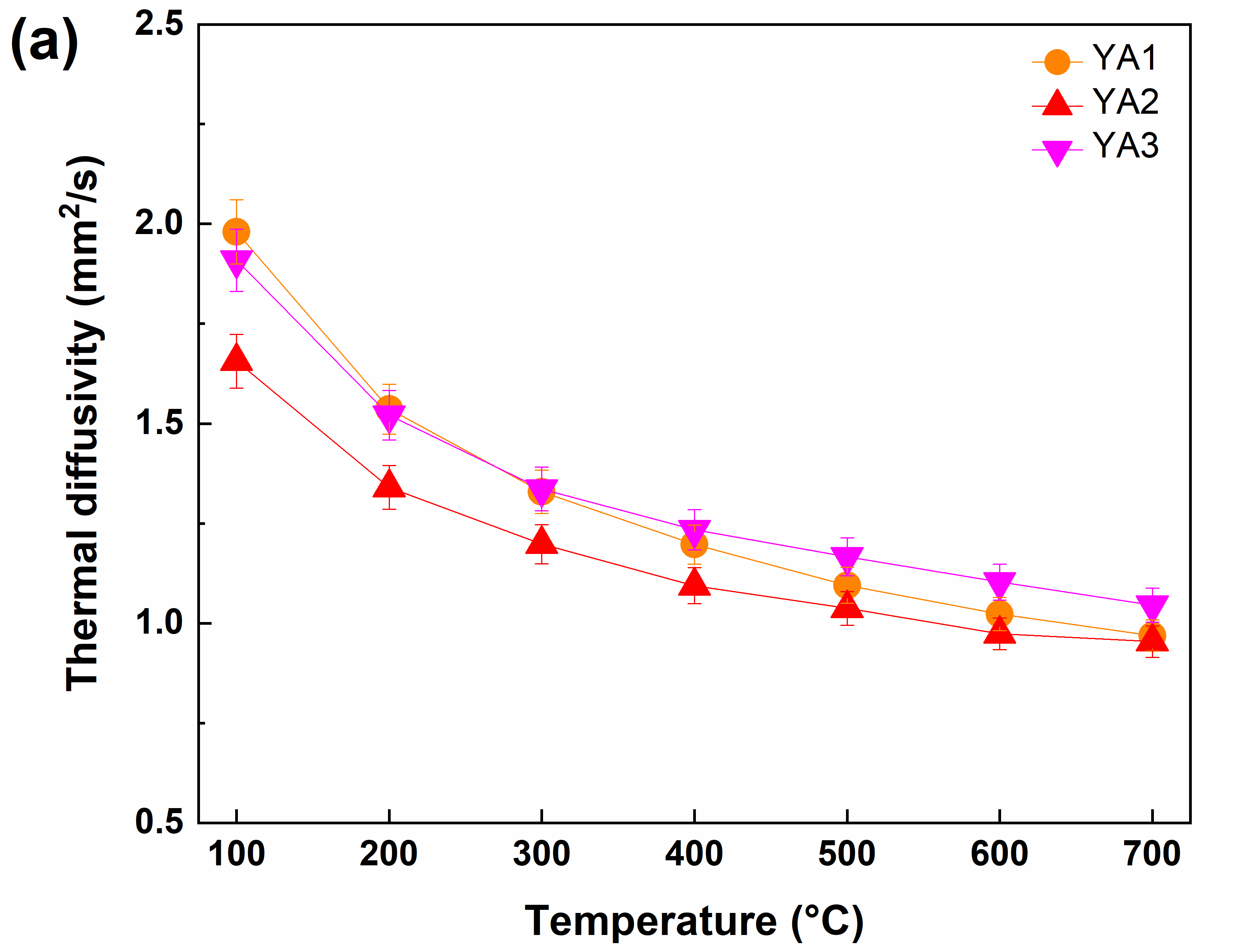

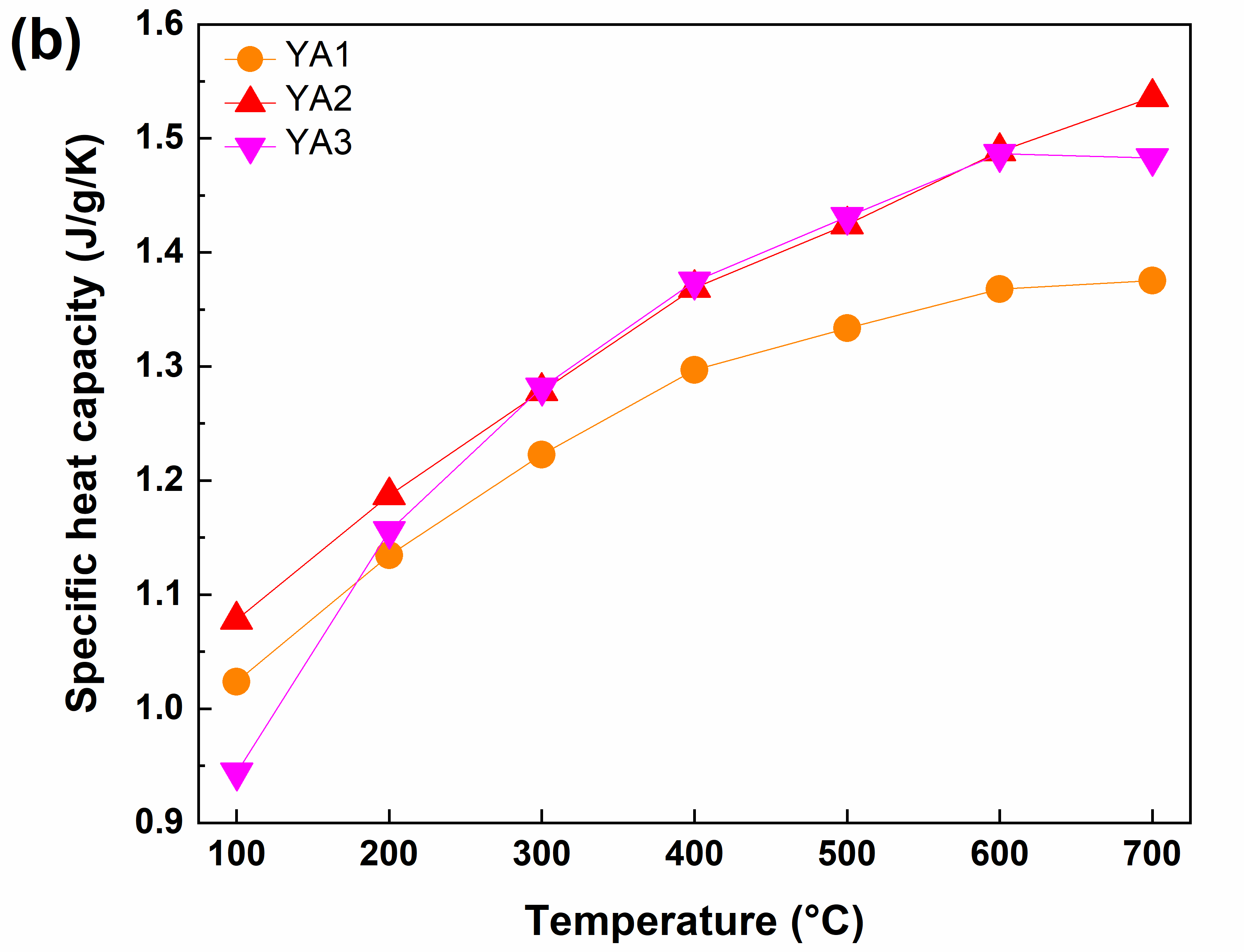


**Figure S1.** (a) Thermal diffusivities and (b) specific heat capacities of n-type Y_x_Al_y_B_14_ (x ~0.64) samples prepared by reactive SPS. Error bars for thermal diffusivities represent a measurement uncertainty range (±5%).
